# Mechanically Reinforced Silkworm Silk Fiber by Hot Stretching

**DOI:** 10.34133/2022/9854063

**Published:** 2022-03-30

**Authors:** Haojie Lu, Kailun Xia, Muqiang Jian, Xiaoping Liang, Zhe Yin, Mingchao Zhang, Huimin Wang, Haomin Wang, Shuo Li, Yingying Zhang

**Affiliations:** Key Laboratory of Organic Optoelectronics and Molecular Engineering of the Ministry of Education, Department of Chemistry, Tsinghua University, Beijing 100084, China

## Abstract

Silkworm silk, which is obtained from domesticated *Bombyx mori* (*B. mori*), can be produced in a large scale. However, the mechanical properties of silkworm silk are inferior to its counterpart, spider dragline silk. Therefore, researchers are continuously exploring approaches to reinforce silkworm silk. Herein, we report a facile and scalable hot stretching process to reinforce natural silk fibers obtained from silkworm cocoons. Experimental results show that the obtained hot-stretched silk fibers (HSSFs) retain the chemical components of the original silk fibers while being endowed with increased *β*-sheet nanocrystal content and crystalline orientation, leading to enhanced mechanical properties. Significantly, the average modulus of the HSSFs reaches 21.6 ± 2.8 GPa, which is about twice that of pristine silkworm silk fibers (11.0 ± 1.7 GPa). Besides, the tensile strength of the HSSFs reaches 0.77 ± 0.13 GPa, which is also obviously higher than that of the pristine silk (0.56 ± 0.08 GPa). The results show that the hot stretching treatment is effective and efficient for producing superstiff, strong, and tough silkworm silk fibers. We anticipate this approach may be also effective for reinforcing other natural or artificial polymer fibers or films containing abundant hydrogen bonds.

## 1. Introduction

Silkworm silk, produced by domesticated silkworm *B. mori*, possesses combined merits such as excellent mechanical properties, renewability, biocompatibility, tunable biodegradability, and industrial-scale production capability [1–3]. Such features make silkworm silk an attractive material not only for traditional textiles [4–6] but also for applications in medical treatment [7], structural materials [8, 9], and smart textiles [10, 11]. Modern industry can produce high-performance synthetic fibers, such as Kevlar and nylon, on a large scale. In comparison, silkworm silk has higher toughness and extensibility than Kevlar fibers and higher stiffness than nylon fibers [1]. However, the tensile strength of silkworm silk is lower than that of Kevlar and nylon. Besides, spider silk is another natural polymer silk which has higher tensile strength, toughness, and extensibility than both silkworm silk and nylon, but it has very limited availability [12, 13]. Therefore, silkworm silk, as a renewable natural material which has great potential for high-performance fibers, attracts great research interests in recent years. Particularly, researchers are constantly exploring effective approaches for reinforcing silkworm silk.

Anisotropic structures play important roles in the tensile properties of fibrous or membranous materials assembled from polymer chains or low-dimensional nanomaterials. For example, natural materials can possess excellent mechanical properties although they only contain simple building blocks, which can be ascribed to their anisotropic architectures [14, 15]. Inspired by the nature, researchers have explored some methods, such as flow-assisted organization [16], directional freeze-casting [17], and spinning with special nozzles or channels [18–20], to optimize the orientation of the building blocks while assembling them into macrofibers or films. The tensile strength or modulus of the obtained materials, such as cellulose fibers [16], polyvinyl alcohol hydrogel [17], graphene fiber [18], MXene fiber [19], and carbon nanotube fiber [20], could be significantly improved. Meanwhile, postprocessing strategies, such as electric current aligning [21] and stretching [22, 23], can also be used to improve the orientation of the building blocks for reinforcing purposes.

Silkworm silk assembled from polymer chains has semicrystalline, oriented, and hierarchical structures [13, 24, 25]. Natural silkworm silk fiber is composed of 20–200 nm thick microfibrils, and the microfibrils are assembled from ~3 nm thick nanofibrils [14, 24, 26]. The silk nanofibrils mainly consist of two kinds of secondary structures: the crystalline structure (*β*-sheet) and the amorphous structure (random coil and *α*-helix) [27]. This kind of unique hierarchical structure endows natural silk fibers with attractive mechanical properties. The *β*-sheet plays an important role in achieving high tensile strength and stiffness of silk fibers while random coil and *α*-helix structures contribute to its extensibility [28, 29]. Researchers have used fast spinning technique to improve the crystalline orientation of silk fibroin to reinforce silk fibers during natural spinning [30, 31] or regenerated spinning [32] processes. Although the above approaches are effective, a more convenient, effective, and easier to be scaled up approach to enhance the mechanical properties of silkworm silk fibers is still lacking. Until now, there have been no reports of directly improving the crystalline orientation of natural silk fibers obtained from silk cocoons to prepare strong silk fibers.

Herein, we report a hot stretching process to reinforce natural silkworm silk through increasing the content and orientation of *β*-sheet nanocrystals of silk fibers, leading to significantly and simultaneously improved stiffness, tensile strength, and toughness. Remarkably, the obtained hot stretched silk fibers (HSSFs) exhibit Young's modulus of 21.6 ± 2.8 GPa, which is much higher than that of pristine silk (11.0 ± 1.7 GPa). The HSSFs also show improved tensile strength of 0.77 ± 0.13 GPa. We observed that both the content and orientation of *β*-sheet nanocrystals increased after the hot stretching process, which contributes to the reinforcement of the fibers. Comparison shows the mechanical performance of the HSSFs is superior to many natural and engineering materials in terms of both stiffness and tensile strength.

## 2. Results

### 2.1. Preparation and Structure Evolution of HSSFs

**Figure 1:**
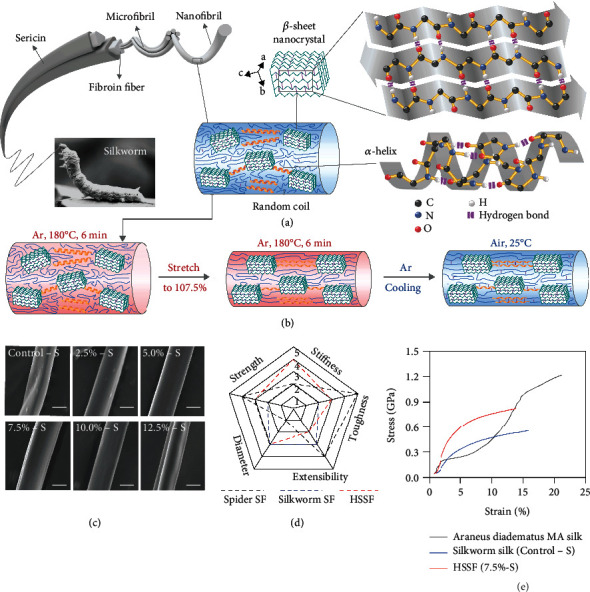
Hierarchical structure, fabrication process, morphology, and mechanical performance of hot-stretched silk fibers (HSSFs). (a) Illustration showing the hierarchical structures and secondary structures of natural silk. (b) Hot stretching process and the secondary structure evolution of the silk fibers. (c) SEM images of Control-S and HSSFs. Scale bar, 5 *μ*m. (d) Performance comparison of the as-obtained HSSFs with *Araneus diadematus* major ampullate (MA) silk fiber (spider SF) and pristine silkworm silk fiber (silkworm SF). (e) Typical stress-strain curves of *Araneus diadematus* MA silk, silkworm silk (Control-S), and HSSF (7.5%-S). The stress-strain curve of *Araneus diadematus* MA silk is redrawn from a reference (33).

Figure 1(a) illustrates the hierarchical structures of silkworm silk fibers, and Figure 1(b) shows the hot stretching process and its effects on the assembling structures of silk fibroins. Degummed silkworm (*B. mori*) silk fibers were used as raw fibers (Control-S). A degummed silk fiber was first heated at 180°C in argon (Ar) for 6 min. Then, the silk fiber was stretched along its axial direction to a certain ratio (2.5%-15.0%) and held for 6 min at 180°C. Finally, the stretched silk fiber was cooled to room temperature in Ar to get the final HSSF. According to the stretch ratio, the obtained HSSFs were denoted as 2.5%-S, 5.0%-S, 7.5%-S, 10.0%-S, 12.5%-S, and 15.0%-S, respectively.

The proposed secondary structure evolution of the silk fibers during the hot stretching process is also illustrated in Figure 1(b). Upon heating, the hydrogen bonds in silk fibers were broken or weakened, enabling the movement of molecular chains in amorphous components. Stretching promotes the alignment of the molecular chain of the amorphous structures and the orientation of *β*-sheets along the axis of the silk fiber. The new structure state can be kept after cooling through forming intermolecular hydrogen bonds between the molecular chains. Ultimately, some of the aligned molecular chains of random coil and *α*-helix are restructured to *β*-sheet nanocrystals, which are mostly aligned along the fiber axis. Therefore, the hot stretching process improves both the content and orientation of *β*-sheet nanocrystals, which can contribute to the improved mechanical properties of HSSFs.

### 2.2. Morphology and Mechanical Properties of HSSFs

In contrast to the pristine silk fibers, the HSSFs show significantly improved uniformity in macroscopic morphology and enhanced stiffness and tensile strength in mechanics.  Figure 1(c) shows typical scanning electron microscopy (SEM) images of Control-S and HSSFs, indicating that the surface of HSSFs is smoother than that of Control-S. The natural silkworm spinning process generally results in nonuniform morphology and varied diameters. The hot stretching provides an opportunity to optimize the pristine structure and morphology of natural fibers. Increasing the stretching ratio leads to a smaller diameter of HSSFs (Figure S4e). The average diameter of 15%-S is 10.54 ± 0.70 *μ*m, smaller than that of Control-S (12.05 ± 0.47 *μ*m). In addition, the hot stretching process endowed HSSFs with better mechanical properties than the pristine silk.  Figure 1(d) compares the mechanical performance of the HSSFs with that of natural silkworm silk fibers and spider silk fibers (details are shown in Tables S1 and S2). In order to show the differences more clearly, the stress-strain curves of different silks are shown in Figure 1(e). Obviously, the HSSFs have excellent comprehensive mechanics (tensile strength, stiffness, and toughness), especially outstanding stiffness (evaluated by Young's modulus).

**Figure 2:**
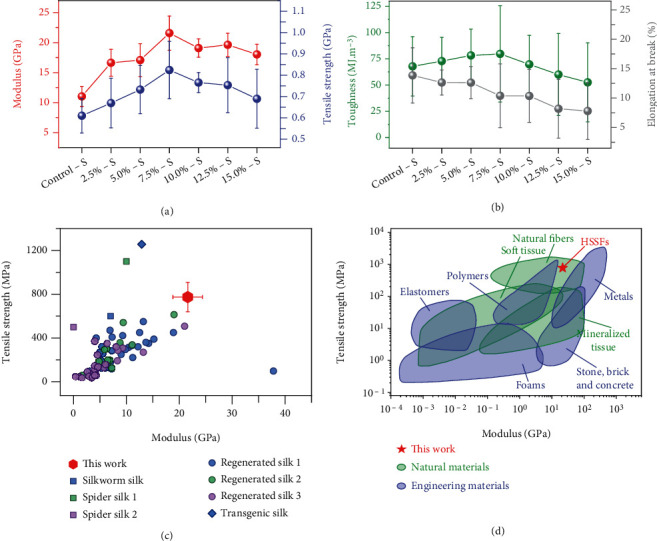
Mechanical properties of the as-obtained silk fibers and performance comparison of HSSFs with other materials: (a, b) Young's modulus, tensile strength, toughness, and elongation at break of different silk fibers; (c) comparison of Young's modulus and tensile strength of HSSFs with natural silk and regenerated silk. “This work” corresponds to 7.5%-S. “Silkworm silk” corresponds to Control-S. Spider silk 1 and 2 correspond to *Araneus diadematus* MA and viscid silk, respectively. Regenerated silk 1, 2, and 3 correspond to regenerated silkworm silk by wet spinning, regenerated silkworm silk by dry spinning, and regenerated natural or recombinant spider silk, respectively. Detailed data and their sources are shown in Table S4. (d) Comparison of Young's modulus and tensile strength of HSSFs with natural and engineering materials. The Ashby plot was drawn according to two references (34, 35).

The mechanical properties of the as-prepared HSSFs are shown in Figures 2(a) and 2(b). According to the stress-strain curves (Figure S1, converted from the load-strain curves in Figure S2), Young's modulus, tensile strength, toughness, and elongation at break of silkworm silk fibers were obtained. We studied the influence of heating time, heating temperature, atmosphere, and stretch ratio on the mechanical properties of the obtained fibers. According to the mechanical properties of different samples (Figures S3 and S4), 12 min and 180°C are selected as the optimized processing parameters. Besides, we compared the mechanical properties of silk fibers prepared with the same conditions except for in Ar or in air (Table S3). The tensile strength, toughness, and elongation at break of silkworm silk prepared in Ar are higher than those of silk prepared in air, indicating Ar plays an important role in protecting silk from being damaged at high temperature.  Figure 2(a) shows the dependence of Young's modulus and tensile strength on the stretch ratio, presenting a strong correlation between them (detailed mechanical properties are shown in Figure S5). Significantly, average Young's modulus of 7.5%-S is up to 21.6 ± 2.8 GPa, compared with 11.0 ± 1.7 GPa of Control-S, indicating the great improvement of stiffness after the hot stretching. With the increase of stretch ratio from 0.0% to 7.5%, the tensile strength of HSSFs increased from 0.56 ± 0.08 GPa to 0.77 ± 0.13 GPa, corresponding to an enhancement of about 40%. It should be noted that the stretching process will consume part of the deformation capacity of the silk molecular chains, resulting in reduced elongation at break. The average elongation at break is reduced from 16.4% of Control-S to 11.9% of 7.5%-S. However, because of the significant improvement of Young's modulus and tensile strength, the HSSF still shows improved toughness (Figure 2(b)). The average toughness of 7.5%-S is 80 ± 46 MJ m^−3^.

The HSSFs show superior mechanical Young's modulus and tensile strength compared with natural and engineering materials.  Figure 2(c) compares the tensile strength and Young's modulus of HSSFs (7.5%-S) with those of natural silkworm silk, natural spider silk, and regenerated silk. Remarkably, the tensile strength and Young's modulus of HSSFs not only exceed those of natural silkworm silk but also are obviously higher than those of various regenerated silks (details are shown in Table S4). Further, Figure 2(d) shows a comparison of the mechanical performance of HSSFs with typical natural materials (natural fibers, soft tissue, and mineralized tissue) and engineering materials (elastomers, polymers, foams, stone, brick, concrete, and metals) reported in literature [34]. It is obvious that Young's modulus and tensile strength of HSSFs are better than those of most natural fibers. Besides, HSSFs are stiffer and stronger than soft tissue and mineralized tissue. Furthermore, compared with engineering materials, HSSFs not only exceed elastomers, most polymers, and foams in Young's modulus and tensile strength but also are stiffer than stone, brick, and concrete and stronger than many metals, making HSSFs potential candidates for structural materials in numerous fields.

### 2.3. Thermal Stability of Silk Fibers

Thermal stability analysis indicates that the chemical components of silk fibers can be maintained during the above thermal treatment process. As shown in Figure S6a-c, the thermogravimetric (TG) curve, differential thermogravimetry (DTG) curve, and thermogravimetric Fourier-transform infrared spectroscopy (TG-FTIR) spectra show that the degummed silkworm silk fibers start to decompose at 230°C in an inert atmosphere. In addition, the pristine silk and the silk treated at 180°C show similar solid state nuclear magnetic resonance (NMR) spectra (Figure S6d), indicating the chemical components of the silk are maintained.

### 2.4. The Content and Orientation of *β*-Sheet Nanocrystals in Silk Fibers

**Figure 3:**
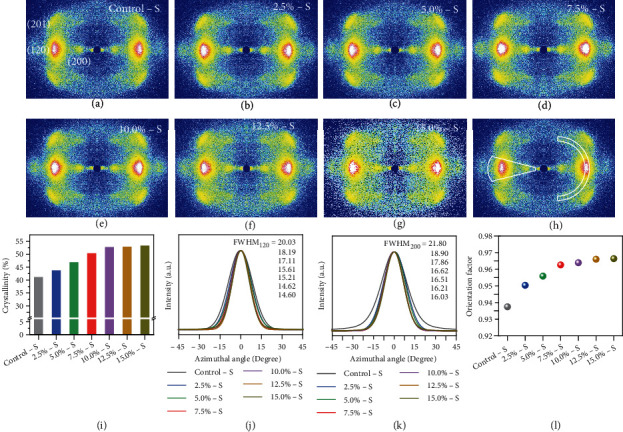
Crystallinity and crystalline orientation of HSSFs: (a–g) WAXS patterns of Control-S and HSSFs; (h) an example showing the integration of WAXS pattern intensity as a function of diffraction angle along the equatorial direction and a function of azimuth angle; (i) crystallinity of different silk fibers; (j, k) 1D azimuthal intensity profiles of the radially integrated (120) and (200) peaks, respectively; (l) orientation factor of different silk fibers.

To investigate the relationship of mechanical properties, secondary structures, and stretch ratio, we studied the structural evolution of HSSFs with an increased stretch ratio, mainly including the crystallinity of HSSFs and the orientation of *β*-sheet nanocrystals. Figures 3(a)–3(g) show the wide-angle X-ray scattering (WAXS) patterns of Control-S and HSSFs prepared with different stretch ratios. The clear and sharp scattering peaks, which correspond to (201), (200), and (120) of silk [36], prove the high crystallinity and high crystalline orientation of the silk fibers. Furthermore, we quantitatively evaluated the crystallinity and crystalline orientation by integration of the intensity of the WAXS patterns (Figure 3(h), Figures S7 and S8). As shown in Figure 3(i), the crystallinity of HSSFs increases with an increased stretch ratio, especially in the stretch ratio range of 2.5%-7.5%, indicating that the hot stretching process promoted the crystallization of the silk fibroin in the fibers. In Control-S, there are more amorphous components than crystalline components. After stretching, parts of the amorphous component are transformed into crystalline structures. When the stretch ratio is above 7.5%, the content of crystalline components is higher than that of amorphous components. The *β*-sheet nanocrystals also show improved alignment along the silk fiber axis. As shown in Figures 3(j) and 3(k), the full width at half maximum (FWHM) of peak (120) and (200) shows obvious decrement with an increased stretch ratio, indicating that the crystalline orientation is increased by stretching. The FWHM tends to be the same for stretch ratios in the range of 7.5%-15.0%, indicating that the orientation is almost the best when the stretch ratio is above 7.5%. The orientation factor was calculated according to the FWHM. As shown in Figure 3(l), the orientation factor of 15.0%-S reaches 0.97, indicating the hot stretching process promotes the alignment of *β*-sheet nanocrystals along the fiber axis.

**Figure 4:**
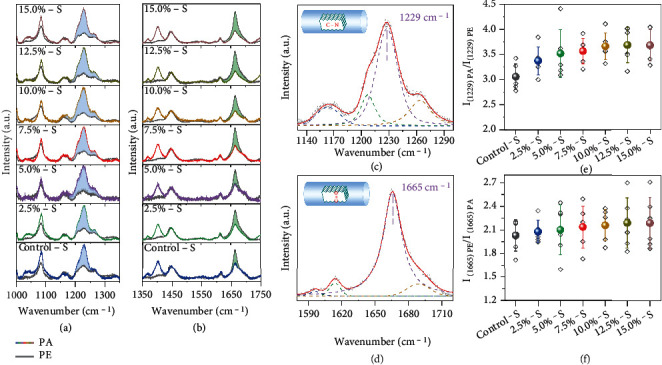
Polarized Raman spectroscopy analysis of HSSFs: (a, b) Raman spectra of silk fibers recorded when silk fibers aligned perpendicular (gray curves) or parallel (colored curves) to the polarization direction of the laser beam; (c, d) an example showing the fitting results of 1229 cm^−1^ and 1665 cm^−1^. The insets show the relative direction of the corresponding chemical bonds to the fiber axis; (e, f) intensity ratios of different silk fibers.

Polarized Raman spectroscopy analysis further proved the improved crystalline orientation of *β*-sheet in the HSSFs compared with the Control-S. As the basic structural unit of silk fibers, the orientation of molecular chains gives insight into the arrangement of microstructures. Figures 4(a) and 4(b) show Raman spectra of the silk fibers in different ranges with the silk fibers aligned perpendicular or parallel to the polarization direction of the laser beam. All the spectra have similar profiles, and there is no obvious change in peak positions, indicating that the chemical composition of the silk fibers is not changed by the hot stretching process. The peaks located at 1229 cm^−1^ and 1665 cm^−1^ are ascribed to the C–N bonds of amide III and C=O bonds of amide I, respectively [37]. The C–N and C=O bonds mainly aligned parallel and perpendicular to the *c*-direction of the *β*-sheet nanocrystals, respectively (see insets of Figures 4(c) and 4(d)). We compared the Raman intensity of C–N and C=O bonds obtained with different polarization directions. For all of the silk fibers, the peak intensity of 1229 cm^−1^ obtained with the fiber parallel to the laser polarization direction (*I*_[1229]PA_) is larger than that obtained with the fiber perpendicular to the laser polarization direction (*I*_[1229]PE_). In contrast, for 1665 cm^−1^, *I*_[1665]PA_ is smaller than *I*_[1665]PE_. These results indicate most of C–N bonds and C=O bonds in molecular chains aligned parallel and perpendicular to the silk fiber axis, respectively. Therefore, most of the *β*-sheet aligned to the fiber axis in silk fibers. As shown in Figures 4(c) and 4(d), the Raman profiles are fitted to obtain the accurate peak intensities of 1229 cm^−1^ and 1665 cm^−1^, respectively (detailed results are shown in Figure S9). Figures 4(e) and 4(f) show the calculated peak intensity ratio of 1229 cm^−1^ and 1665 cm^−1^ obtained in different directions. It can be observed that both *I*_[1229]PA_/*I*_[1229]PE_ and *I*_[1665]PE_/*I*_[1665]PA_ increase with the increased stretch ratio from 0% to 15.0%, indicating that the crystalline orientation is progressively enhanced by the hot stretching process.

**Figure 5:**
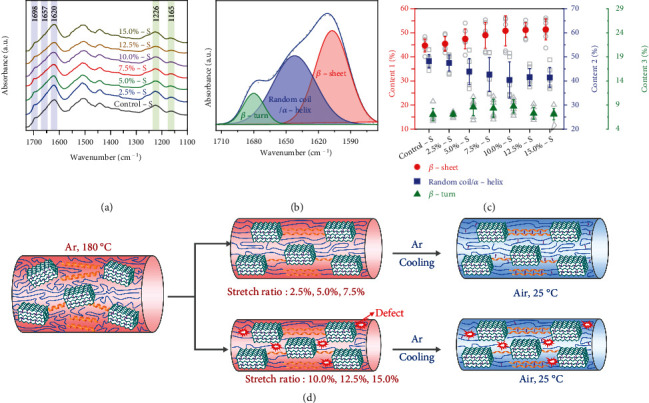
FTIR spectroscopy analysis and illustration showing the structure evolution of the fiber under hot stretching: (a) FTIR spectra of HSSFs; (b) an example showing deconvolution of FTIR spectra in amide I band of HSSFs; (c) contents of *β*-sheet, random coil/*α*-helix, and *β*-turn in different silk fibers; (d) illustration showing the proposed evolution of the secondary structures for relatively small and large stretch ratios.

Fourier transform infrared spectroscopy (FTIR) further revealed the structure transformation from random coil/*α*-helix to *β*-sheet induced by the hot stretching process.  Figure 5(a) shows the FTIR spectra of silk fibers with different stretch ratios. FTIR bands corresponding to amide I (1580-1720 cm^−1^) and amide III (1200-1300 cm^−1^) can be clearly observed. To calculate the contents of different secondary structures, the amide I band of silk was deconvoluted (detailed results are shown in Figure S10). As shown in Figure 5(b), there are three peaks located at 1620, 1657, and 1698 cm^−1^, which can be assigned to *β*-sheet, random coil/*α*-helix, and *β*-turn [38, 39], respectively.  Figure 5(c) shows the calculated contents of the three kinds of secondary structures. The content of *β*-sheet increases with increasing stretch ratio, while the content of random coil/*α*-helix continuously decreases, which is consistent with the results of WAXS. It should be noted that in a reported work [40], the content of *β*-sheet decreased with increased temperature, which is different from this work. We propose that although the heating results in a reduction of *β*-sheet, the stretching leads to increased *β*-sheet, and it seems that the effect of stretching is stronger than the effect of heating in our results.

Figure 5(d) illustrates the proposed structure evolution of HSSFs under the hot stretching process. According to WAXS, Raman, and FTIR analysis, the crystallinity (content of *β*-sheet nanocrystals) and crystalline orientation increase obviously in relatively small stretch ratios (2.5%, 5.0%, and 7.5%), while tending to be constant in relatively large stretch ratios (10.0%, 12.5%, and 15.0%). We proposed that, for small stretch ratios, the hierarchical structures of silk possess enough deformation capacity, enabling enhanced crystallization with improved alignment without damaging the structure. When the stretch ratio is over 7.5%, there is almost no more deformation capacity for silk fibers, and further stretch will result in the formation of defects, which may lead to deteriorative properties.

### 2.5. Potential Applications of HSSFs

The combined high strength, high toughness, and light weight of HSSF enable it to be a structural material with many potential applications. The as-obtained HSSFs show a high strength of 10.4 ± 1.8 cN/dtex (taking the density of silkworm silk as 1.35 g cm^−3^ for unit conversion [41]) and a high toughness of 80 MJ m^−3^. Due to the combination of high mechanical performance and light weight, HSSFs can be used in fabricating strong cables or fabrics to replace Kevlar or spider silk in some cases. Although Kevlar and spider silk have superior performance [42], they are facing some challenges. For example, although Kevlar is strong and stiff, its toughness is not ideal for effectively protecting the human body. For natural spider silk, its manufacture is a great challenge. Therefore, considering the large-scale production potential of HSSFs, it may become a new candidate for strong fibers.

For proof of concepts, we demonstrated the applications of the HSSFs in several scenarios where strong cables are required. We observed that a HSSF yarn containing 35 fibers can easily hang a model spider man (~59 g) attached with a flying toy helicopter (Supplementary Movie S1) and pull up a box of stones (156 g) with a model tower crane (Supplementary Movie S2). Moreover, a running toy car can be stopped by a HSSF cable due to the reverse traction (Supplementary Movie S3). The high toughness enables the HSSFs to absorb the energy of the car as it moves forward, and the high tensile strength and Young's modulus allow the HSSFs to hold the car without breakage. Besides, the HSSFs may be used as the cable of a sailboat (Supplementary Movie S4).

Besides, HSSF may serve as lightweight reinforcement of structural materials applied in wind turbine blades, aircraft wings, and protective shields, which need to bear large forces under extreme conditions. Previous work has proved that silk could effectively strengthen and toughen resinous materials to prepare impact-critical structural composites [8]. We proposed that the HSSF with further enhanced mechanical properties may be a better candidate for these applications compared to the pristine silks.

## 3. Discussion

In summary, we report a hot stretching strategy to produce reinforced natural silk. WAXS and FTIR results show that the hot stretching process promotes the transformation of secondary structures of silk fibroin from random coil/*α*-helix to *β*-sheet. At the same time, the crystalline orientation in the silk fiber is also enhanced by the hot stretching. Improved crystallinity and enhanced crystalline alignment lead to superior mechanical properties of HSSFs. Tensile tests prove that HSSFs possess simultaneously improved Young's modulus, tensile strength, and toughness. Remarkably, the as-obtained HSSFs showed average Young's modulus of 21.6 ± 2.8 GPa, which is about twice that of pristine silkworm silk fibers (11.0 ± 1.7 GPa). The average tensile strength and toughness of the HSSFs reach 0.77 ± 0.13 GPa and 80 ± 46 MJ m^−3^, respectively. In addition, mechanical comparison shows that HSSFs are stiffer and stronger than most other natural materials and engineering materials. Finally, we discussed the potential applications of the HSSFs where strong and lightweight fibers are required. This hot stretching process is facile and green and has potential to produce superior fibers on a large scale, making the obtained HSSF a promising candidate for advanced structural materials.

## 4. Materials and Methods

### 4.1. Degumming of Silk Fibers

The *B. mori* silkworm cocoon was boiled in an aqueous solution of NaHCO_3_ (0.5% *w*/*w*) for 20 minutes and rinsed five times with deionized water, which was repeated twice to remove the sericin of the silk. After degumming, the silk fibers were dried at 60°C for 12 hours to obtain degummed silk fibers.

### 4.2. Preparation of HSSFs

Aligned degummed silk fibers were fixed on the platform of an internal measuring micrometer. The micrometer was placed in the hot zone filled with Ar (purity, 99.999%) of a quartz tube heated with an electrical thermal belt and kept for 6 minutes. The heated silk fibers were stretched to a certain ratio by tuning the micrometer. After 6 minutes, the fibers on the micrometer were naturally cooled to room temperature in Ar to obtain HSSFs. The above experimental parameters, such as heating time, temperature, and stretch ratio, were optimized (details are shown in supporting information).

### 4.3. Spectroscopy Characterization

WAXS was carried out to investigate the crystallinity of Control-S and HSSFs and the orientation factor of *β*-sheet nanocrystals. We winded more than 50 silk fibers prepared under the same conditions on a sample rack to carry out the test for each sample. The right half of WAXS patterns (Figure 3) was obtained by a WAXS system (XEUSS, Xenocs) instrument and used for calculating crystallinity and the orientation factor, and the complete patterns in Figure 3 were obtained by mirroring. Software Fit 2D and Peak Fit 4 were used to process the patterns to obtain the crystallinity and orientation factor of Control-S and HSSFs. Detailed analysis is shown in supporting information. The Raman spectra were collected with a Raman spectrum instrument (LabRAM HR Evolution, HORIBA Jobin Yvon) using a 633 nm laser. For each single fiber, 4 spectra were recorded to calculate its intensity ratios. For each group, intensity ratios of 6 HSSFs were used to calculate the average intensity ratios. The FTIR spectra were recorded with FTIR spectrometry (PerkinElmer, Spotlight 400). For each group, 6 spectra from 6 HSSFs were recorded for deconvolution to calculate the average content of secondary structures. The solid-state NMR spectra of the pristine silkworm silk and the thermally treated silk were recorded using a JNM-ECZ600R spectrometer equipped with a 3.2 mm probe. The cross-polarization (CP) experiments were performed with a frequency of 150 MHz, a magic angle spinning (MAS) frequency of 12 kHz, and a recycle delay of 3.0 s for 1200 scans.

### 4.4. Thermal Characterization

The TG curve of degummed natural silkworm silk was recorded using a TGA/DSC simultaneous thermal analyzer (Mettler-Toledo, TGA/DSC1/1600LF) to evaluate the thermal stability of silkworm silk. The temperature range was 30-800°C, and the heating rate was 10°C/min in Ar atmosphere. TG-FTIR spectra of degummed natural silkworm silk were recorded using a combined thermal analysis system (NETZSCH, X70). The temperature range was 50-900°C, and the heating rate was 10°C/min in Ar atmosphere.

### 4.5. Diameter and Morphology Characterization

The diameters of Control-S and HSSFs were measured by optical microscopy (Leica, DM2500M). For each single HSSF fiber, five values were recorded to calculate the average diameter. SEM (JEOL, JSM-IT300, 10 kV) was used to observe the morphology of Control-S and HSSFs which were coated with platinum.

### 4.6. Mechanical Tests

A mechanical testing machine (Shimadzu, AGS-X, 5 N load cell, Grade 1%, assurance range 1/500–1/1) was employed to assess the mechanical properties of Control-S and HSSFs at ambient conditions (~20°C, ~25% relative humidity). For each group, 10 values of 10 silk fibers were obtained for the average value and error bar. The gauge length of HSSF was 10 mm, and the tensile rate was 1 mm/min. The load-strain curve for each monofilament sample was obtained. Optical microscopy was used to measure the average diameter of each monofilament sample, and its cross-sectional area was calculated, assuming the cross-section is circular. The load-strain curves were converted to stress-strain curves to obtain Young's modulus, tensile strength, toughness, and elongation at break.

### 4.7. Demonstration of HSSFs

A yarn containing 35 fibers was used for the demonstration of HSSFs (5.0%-S) (Supplementary Movies S1–S4). The radio-controlled helicopter (YD-615), tower crane (NO.567-19), and sports car (D829) were produced by Guangdong Attop Technology Co., Ltd, Shantou Chenghai 567 Toy Factory, Jianjian Technology Co., Ltd, respectively. The original cables of the tower crane and sailboat were replaced by the HSSFs.

## Supplementary Materials

Supplementary Materials The supporting information includes methods for preparation of HSSFs. Analysis of crystallinity and crystalline orientation of HSSFs. Figures and figure captions: Figure S1: stress-strain curves of Control-S and HSSFs with different stretch ratios. Figure S2: load-strain curves of Control-S and HSSFs with different stretch ratios. Figure S3: mechanical properties of silk fibers prepared at different heating times and temperatures. Figure S4: detailed mechanical properties of silk fibers under different heating times and temperatures. Figure S5: detailed mechanical properties of Control-S and silk fibers with different stretch ratios. Figure S6: thermal stability of silkworm silk. Figure S7: 1D radial integration of intensity profiles of integrated (200)/(120) peaks from the WAXS patterns. Figure S8: 1D intensity profile as a function of azimuth angle at the integrated (120) or (200) peaks of different silk fibers. Figure S9: examples showing fitting and deconvolution of Raman spectra. Figure S10: examples showing fitting and deconvolution of amide I band in FTIR spectra. Tables and table captions: Table S1: the standard for classifying the mechanical performance of silk fibers in Figure 1(d). Table S2: comparison of modulus, tensile strength, toughness, and elongation at break of silk fibers. Table S3: mechanical properties of silk prepared in Ar and in air with all other conditions the same. Table S4: modulus and tensile strength of regenerated silk and natural silk in Figure 2(c). Supplementary Movie S1: HSSFs tied to a helicopter to hang a spider man model. Supplementary Movie S2: hot-stretched silk assembled to a tower cane for pulling up and down a basket of stones. Supplementary Movie S3: the skidding of toy sports car due to the reverse traction provided by HSSF. Supplementary Movie S4: the sailing of a sailboat using HSSFs as cables.
