# Supplementary material for: Mechanically Reinforced Silkworm Silk Fiber by Hot Stretching
Source: Research (Wash D C). 2022 Mar 30;2022:9854063. doi: 10.34133/2022/9854063 (PMC8992573; doi:10.34133/2022/9854063)
Supplement: Supplementary Materials — The supporting information includes methods for preparation of HSSFs. Analysis of crystallinity and crystalline orientation of HSSFs. Figures and figure captions: Figure S1: stress-strain curves of Control-S and HSSFs with different stretch ratios. Figure S2: load-strain curves of Control-S and HSSFs with different stretch ratios. Figure S3: mechanical properties of silk fibers prepared at different heating times and temperatures. Figure S4: detailed mechanical properties of silk fibers under different heating times and temperatures. Figure S5: detailed mechanical properties of Control-S and silk fibers with different stretch ratios. Figure S6: thermal stability of silkworm silk. Figure S7: 1D radial integration of intensity profiles of integrated (200)/(120) peaks from the WAXS patterns. Figure S8: 1D intensity profile as a function of azimuth angle at the integrated (120) or (200) peaks of different silk fibers. Figure S9: examples showing fitting and deconvolution of Raman spectra. Figure S10: examples showing fitting and deconvolution of amide I band in FTIR spectra. Tables and table captions: Table S1: the standard for classifying the mechanical performance of silk fibers in Figure 1(d). Table S2: comparison of modulus, tensile strength, toughness, and elongation at break of silk fibers. Table S3: mechanical properties of silk prepared in Ar and in air with all other conditions the same. Table S4: modulus and tensile strength of regenerated silk and natural silk in Figure 2(c). Supplementary Movie S1: HSSFs tied to a helicopter to hang a spider man model. Supplementary Movie S2: hot-stretched silk assembled to a tower cane for pulling up and down a basket of stones. Supplementary Movie S3: the skidding of toy sports car due to the reverse traction provided by HSSF. Supplementary Movie S4: the sailing of a sailboat using HSSFs as cables. [file 9854063.f1.zip › Supplementary Information.docx]

**Supplementary Information**

**Mechanically reinforced silkworm silk fiber by hot stretching**

*Haojie Lu, Kailun Xia, Muqiang Jian, Xiaoping Liang, Zhe Yin, Mingchao Zhang, Huimin Wang, Haomin Wang, Shuo Li, and Yingying Zhang*^*^

Key Laboratory of Organic Optoelectronics and Molecular Engineering of the Ministry of Education, Department of Chemistry, Tsinghua University, Beijing 100084, P. R. China

The supporting information includes:

Methods

Preparation of HSSFs.

Analysis of Crystallinity and Crystalline Orientation of HSSFs.

Figures and Figure Captions

Figure S1. Stress-strain curves of Control-S and HSSFs with different stretch ratios.

Figure S2. Load-strain curves of Control-S and HSSFs with different stretch ratios.

Figure S3. Mechanical properties of silk fibers prepared at different heating time and temperature.

Figure S4. Detailed mechanical properties of silk fibers under different heating time and temperature.

Figure S5. Detailed mechanical properties of Control-S and silk fibers with different stretch ratios.

Figure S6. Thermal stability of silkworm silk.

Figure S7. 1D radial integration of intensity profiles of integrated (200)/(120) peaks from the WAXS patterns.

Figure S8. 1D intensity profile as a function of azimuth angle at the integrated (120) or (200) peaks of different silk fibers.

Figure S9. Examples showing fitting and deconvolution of Raman spectra.

Figure S10. Examples showing fitting and deconvolution of amide I band in FTIR spectra.

Tables and Table Captions

Table S1. The standard for classifying the mechanical performance of silk fibers in Figure 1d.

Table S2. Comparison of modulus, tensile strength, toughness and elongation at break of silk fibers.

Table S3. Mechanical properties of silk prepared in Ar and in air with all other conditions the same.

Table S4. Modulus and tensile strength of regenerated silk and natural silk in Figure 2c.

References

**Methods**

*Preparation of HSSFs.*

To adjust heating time, the heating temperature and stretch ratio are kept at 180 °C and 5%, and the total heating time was set as 4, 12, 20 minutes, and the as-obtained samples were named 4 min-S, 12 min-S and 20 min-S, respectively. When adjusting heating temperature, the total heating time and stretch ratio were kept at 12 min and 5%, and the heating temperature was adjusted to 161, 173, 180, 187, and 199 °C, which were named 161 °C-S, 173 °C-S, 180 °C-S, 187 °C-S, and 199 °C-S, respectively. It is noted that the groups 12 min-S and 180 °C-S are the same as 5.0%-S.

*Analysis of Crystallinity and Crystalline Orientation* *of HSSFs.*

*Crystallinity (C)*: The 1D radial integration of intensity profile of integrated (200)/(120) peaks was deconvoluted into three crystalline peaks and an amorphous peak. The crystallinity was obtained by the ratio of the area of crystalline peaks to the whole area under the curve, which is demonstrated in the following equation:

| $C=\frac{I_{c}}{I_{c}+I_{a}}\times100\%$ | (1) |
| --- | --- |

Here, *I_c_* is the total area of crystalline peaks, and *I_a_* is the area of amorphous peak.

*Orientation factor (f)***:** Herman’s orientation factor *f* is usually employed to evaluate the alignment of crystallites in the axis direction of silk fiber. If *f* = 1, it demonstrates the β-sheet crystallites align completely along the axis of silk fibers. If *f* = 0, it indicates that they distribute randomly in silk fibers. *f* can be calculated from following equations:

|  | (2) |
| --- | --- |

|  | (3) |
| --- | --- |
| $\cos^{2}\theta_{200}=1-\left[\cos\left(0.4FWHM_{200}\right)\right]^{2}$ | (4) |
| $\cos^{2}\theta_{120}=1-\left[\cos\left(0.4FWHM_{120}\right)\right]^{2}$ | (5) |

*φ* describes the angle between the c axis of crystallites and axis of silk fibers. FWHM_200_ and FWHM_120_ and are the full width at half maximum of (200) and (120) peaks, which can be obtained from the corresponding azimuth profiles of the integrated (200) and (120) peaks, respectively.

**Figures and Figure Captions**


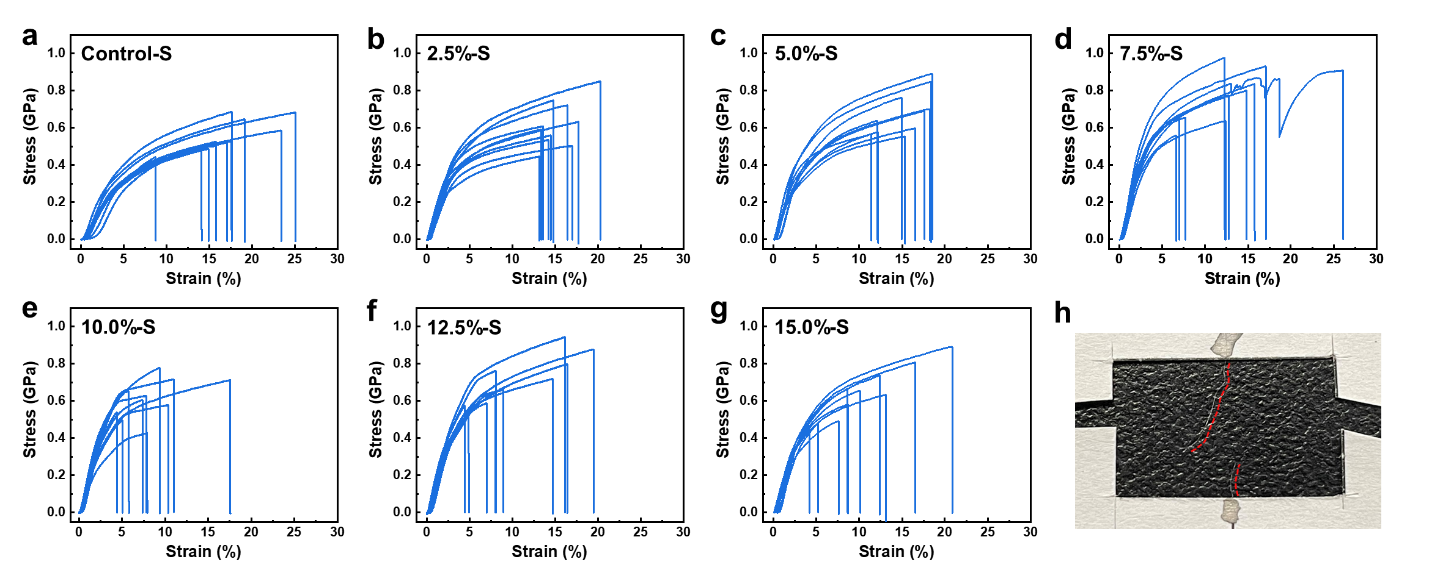


**Figure S1.** Stress-strain curves of Control-S and HSSFs with different stretch ratios. (a) Control-S. (b) 2.5%-S. (c) 5.0%-S. (d) 7.5%-S. (e) 10.0%-S. (f) 12.5%-S. (g) 15.0%-S. (h) A photo of a sample after tensile test, and the white part close to the red dotted line is the broken silk fiber.


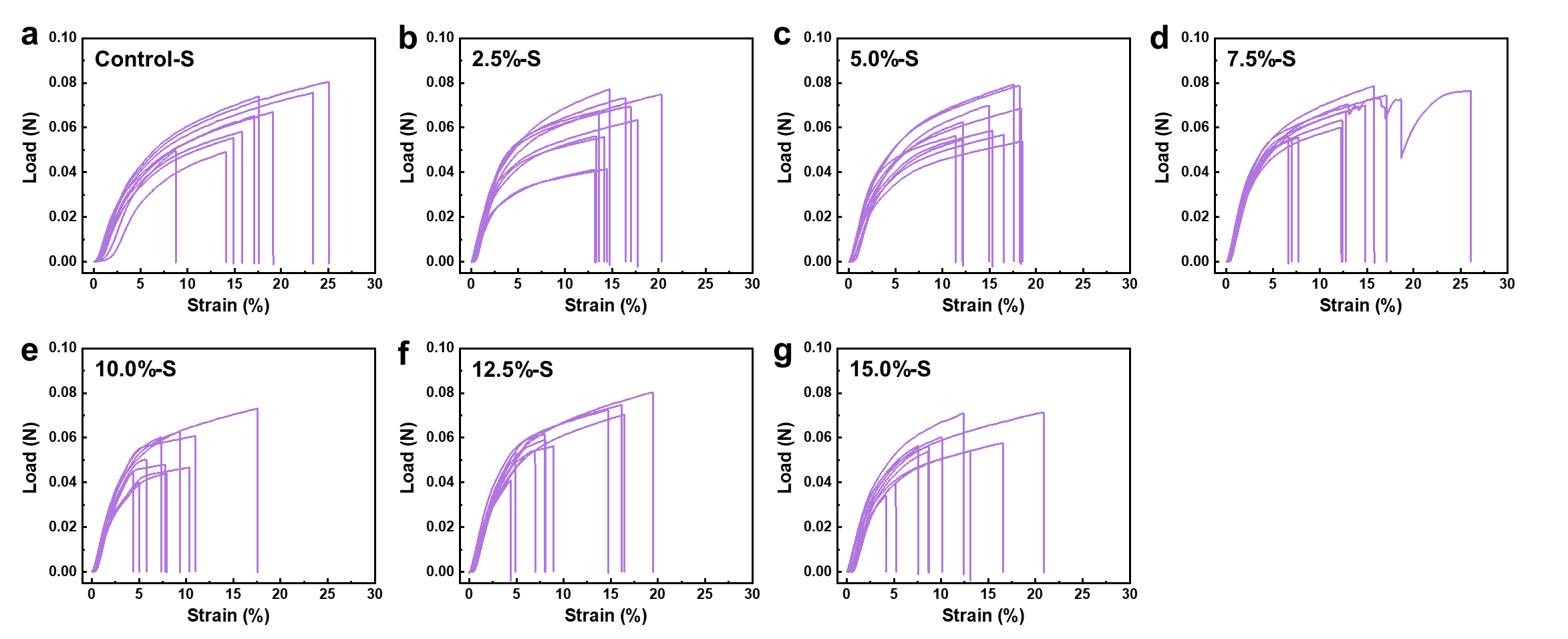


**Figure S2.** Load-strain curves of Control-S and HSSFs with different stretch ratios. (a) Control-S. (b) 2.5%-S. (c) 5.0%-S. (d) 7.5%-S. (e) 10.0%-S. (f) 12.5%-S. (g) 15.0%-S.


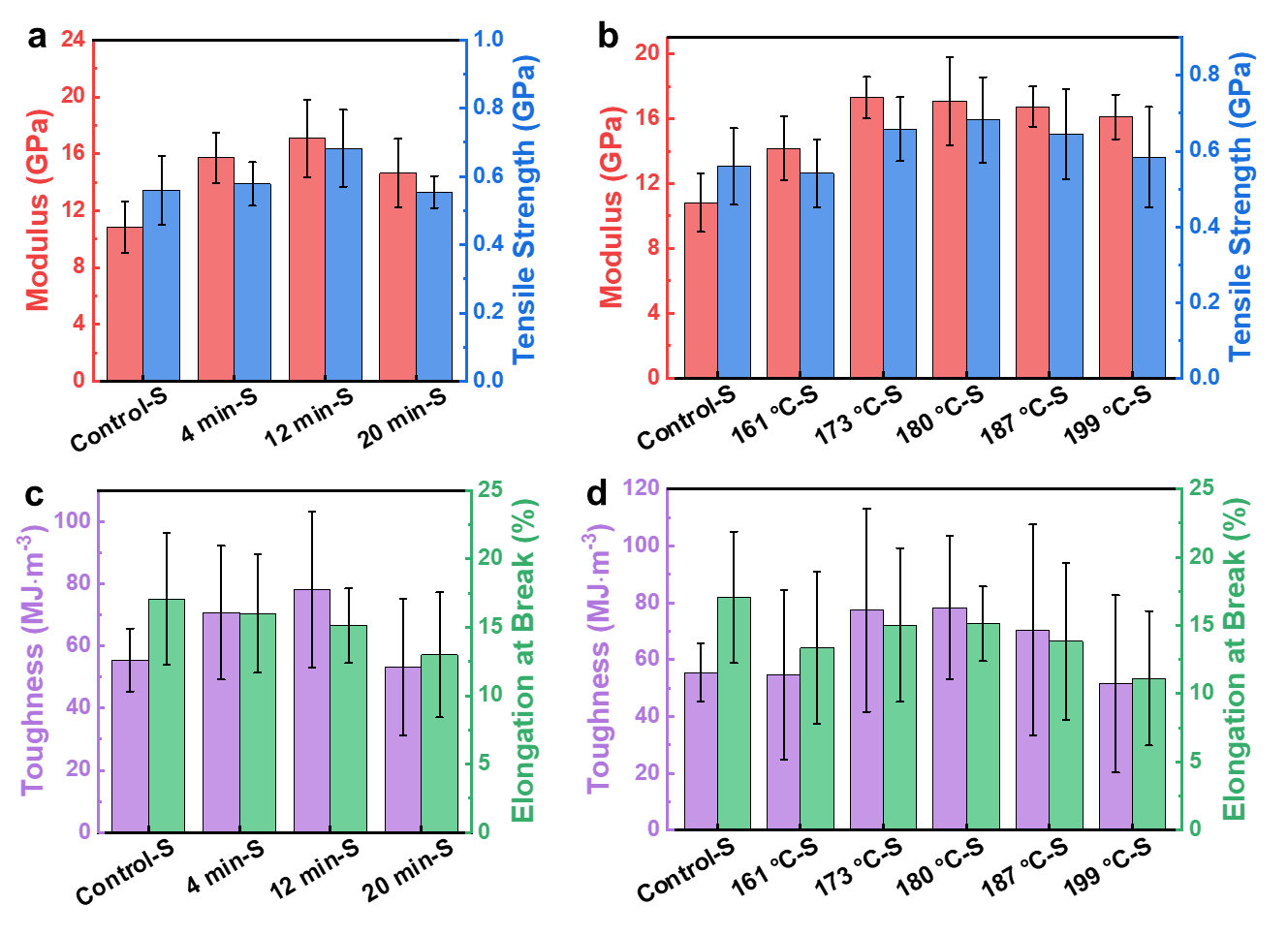


**Figure S3.** Mechanical properties of silk fibers prepared at different heating time and temperature. Average modulus and tensile strength of silks obtained at different heating time (a) and temperature (b). Average toughness and elongation at break of silks obtained at different heating time (c) and temperature (d).


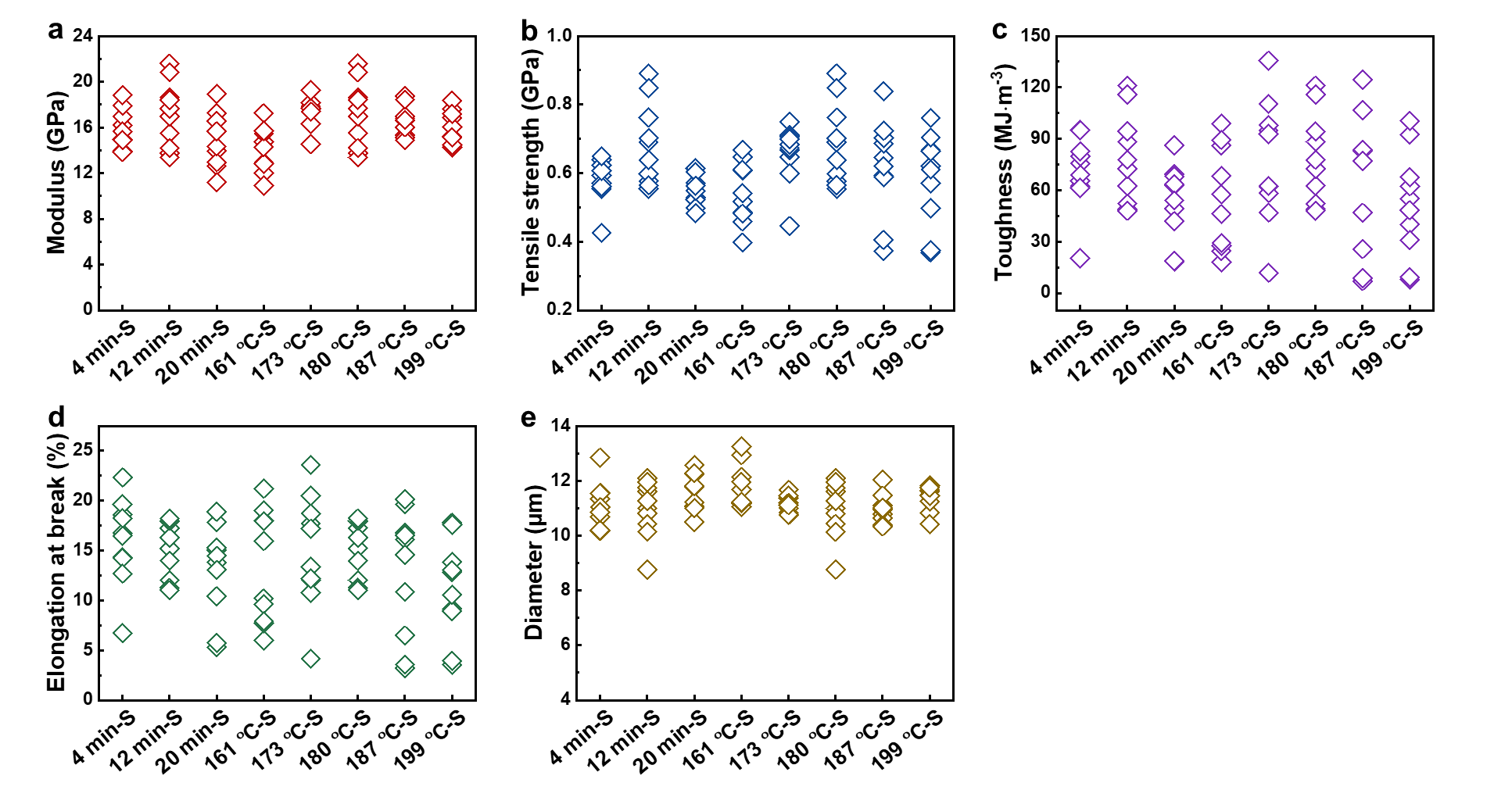


**Figure S4.** Detailed mechanical properties of silk fibers under different heating time and temperature. (a) Young’s modulus. (b) Tensile strength. (c) Toughness. (d) Elongation at break. (e) diameter.


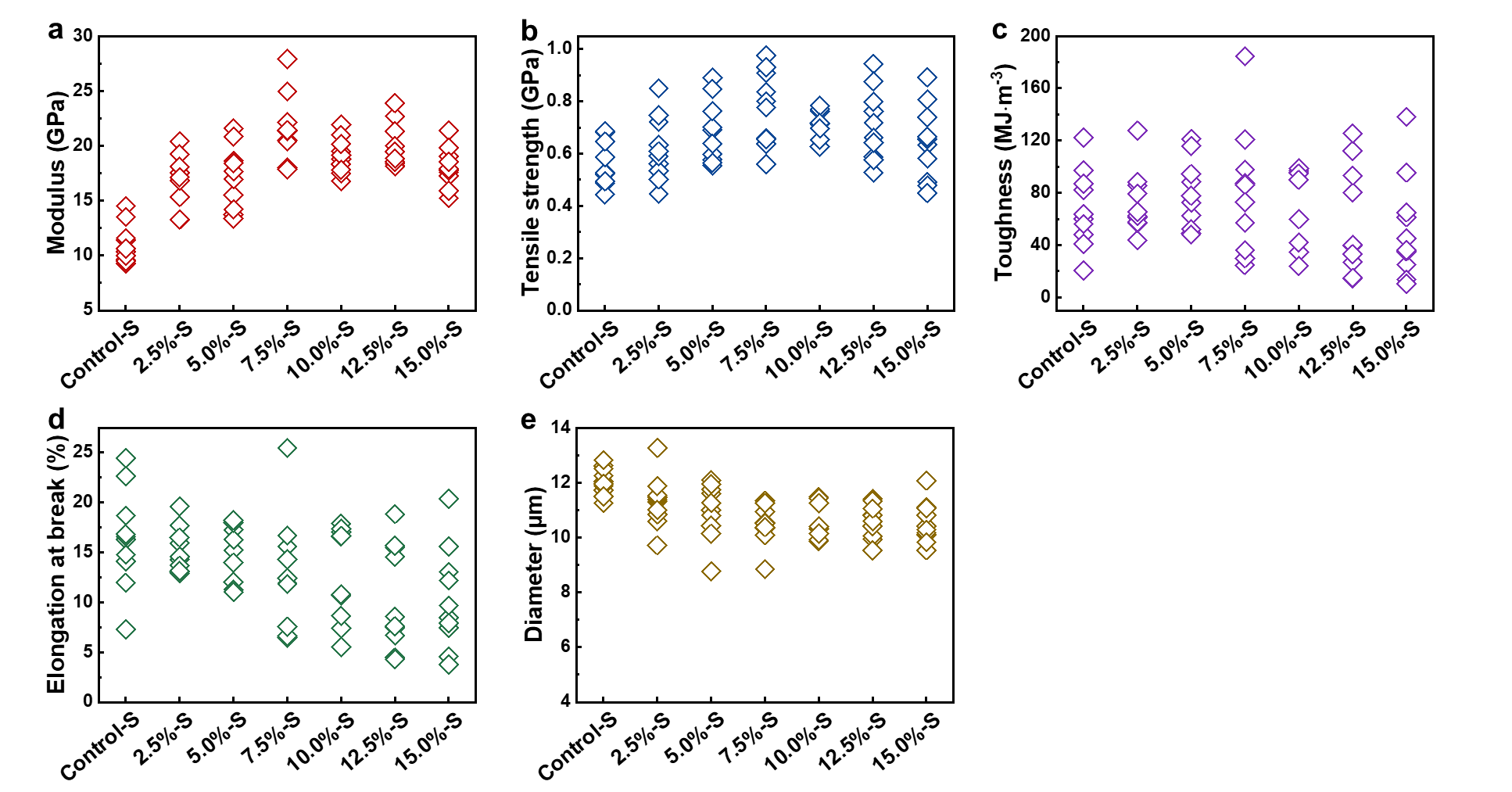


**Figure S5.** Detailed mechanical properties of Control-S and silk fibers with different stretch ratios. (a) Young’s modulus. (b) Tensile strength. (c) Toughness. (d) Elongation at break. (e) diameter.


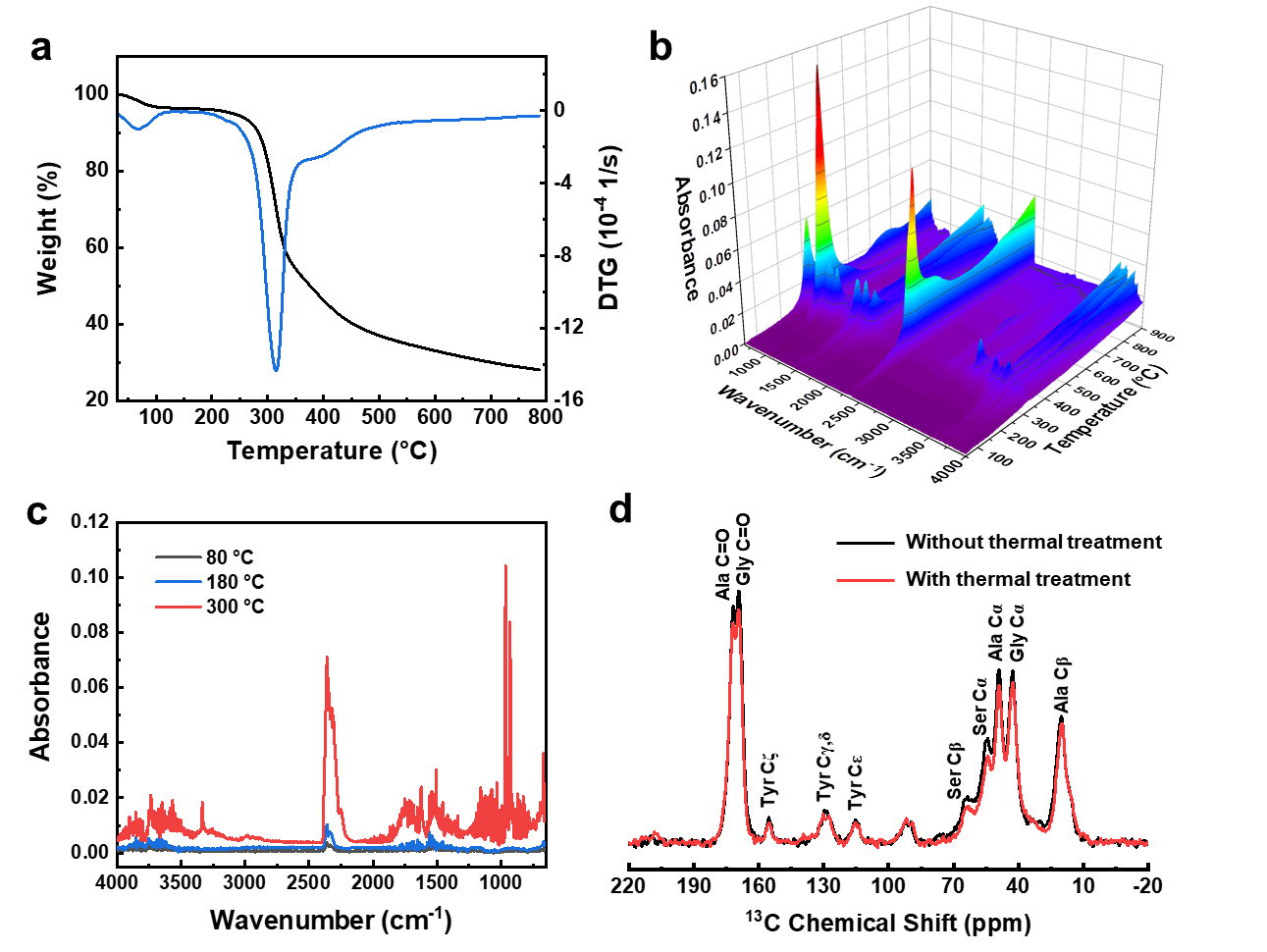


**Figure S6.** Thermal stability of silkworm silk. (a) Thermogravimetric curve and differential thermogravimetry curve of degummed silkworm silk fibers. (b-c) Evolution of thermogravimetric Fourier-transform infrared spectroscopy (TG-FTIR) spectrum with temperature (b) TG-FTIR spectra at 80, 180, 300 °C (c) of degummed silkworm silk fibers. (d) ^13^C cross polarization-magic angle spinning (CP-MAS) spectra of silkworm silk without/with thermal treatment.


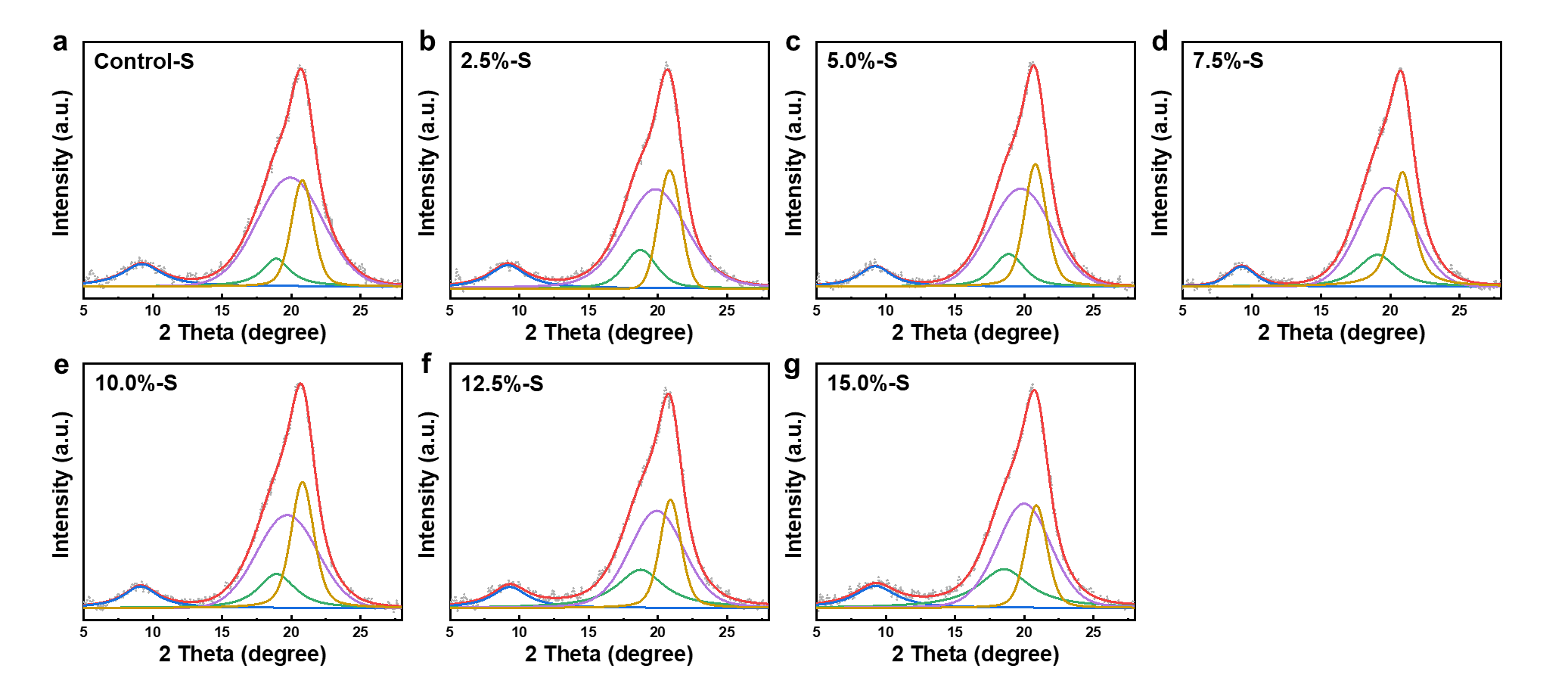


**Figure S7.** 1D radial integration of intensity profiles of integrated (200)/(120) peaks from the WAXS patterns. The plots show the original curves (gray, dotted lines) and the fitting lines (red lines). Each fitting line is deconvoluted into three crystalline peaks (blue, green and olive lines) and an amorphous peak (purple line), which correspond to the (100), (200), (120) Bragg reflections and amorphous component, respectively. (a) Control-S. (b) 2.5%-S. (c) 5.0%-S. (d) 7.5%-S. (e) 10.0%-S. (f) 12.5%-S. (g) 15.0%-S.


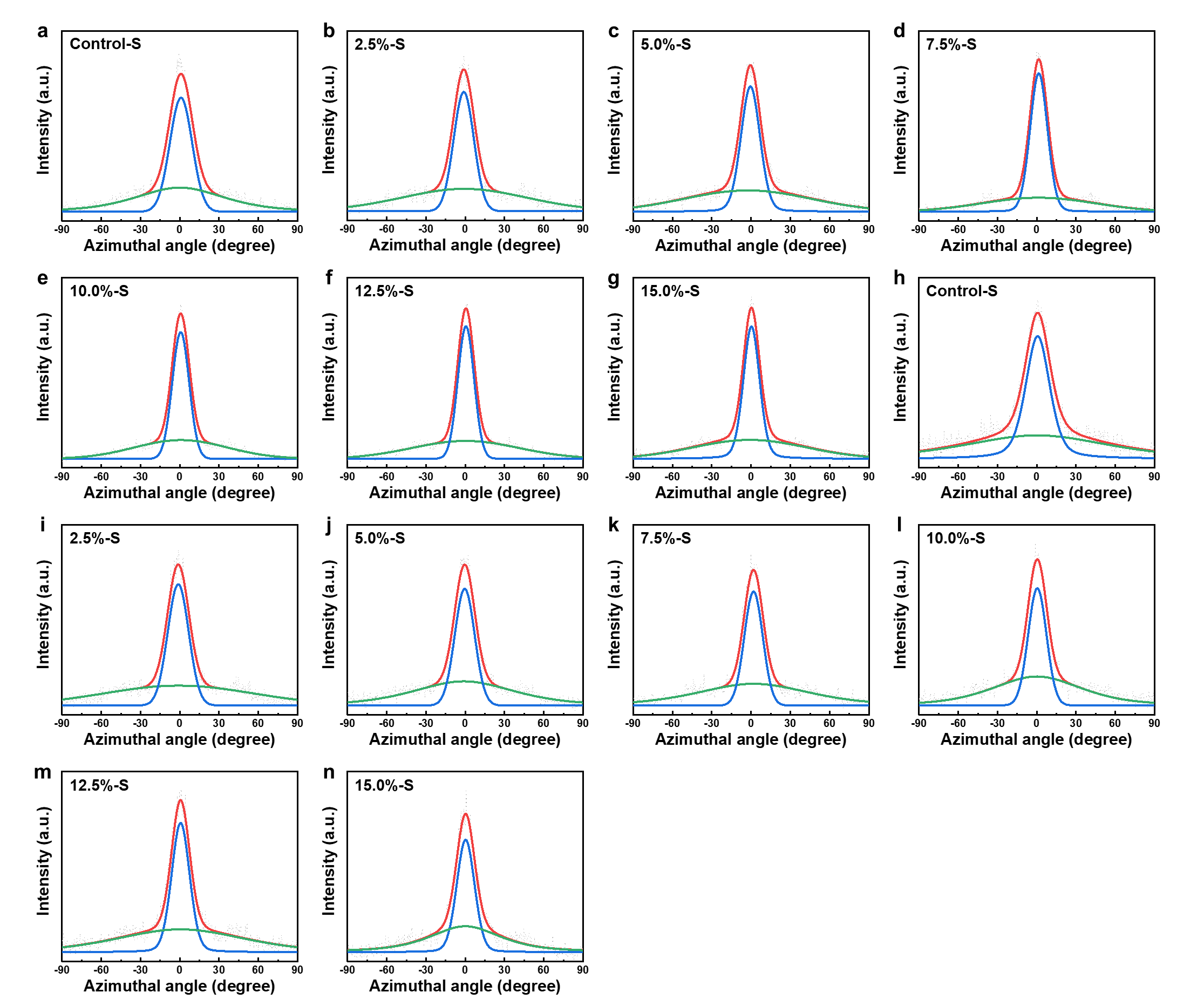


**Figure S8.** 1D intensity profile as a function of azimuth angle at the integrated (120) or (200) peaks of different silk fibers. The plots show the original curves (gray, dotted lines) and the fitting lines (red lines). Each fitting line was deconvoluted to a crystalline peak (blue line) and an amorphous peak (green line). 1D intensity profiles at peak (120) of Control-S (a), 2.5%-S (b), 5.0%-S (c), 7.5%-S (d), 10.0%-S (e), 12.5%-S (f), and 15.0%-S (g), respectively. 1D intensity profiles at peak (200) of Control-S (h), 2.5%-S (i), 5.0%-S (j), 7.5%-S (k), 10.0%-S (l), 12.5%-S (m), and 15.0%-S (n), respectively.


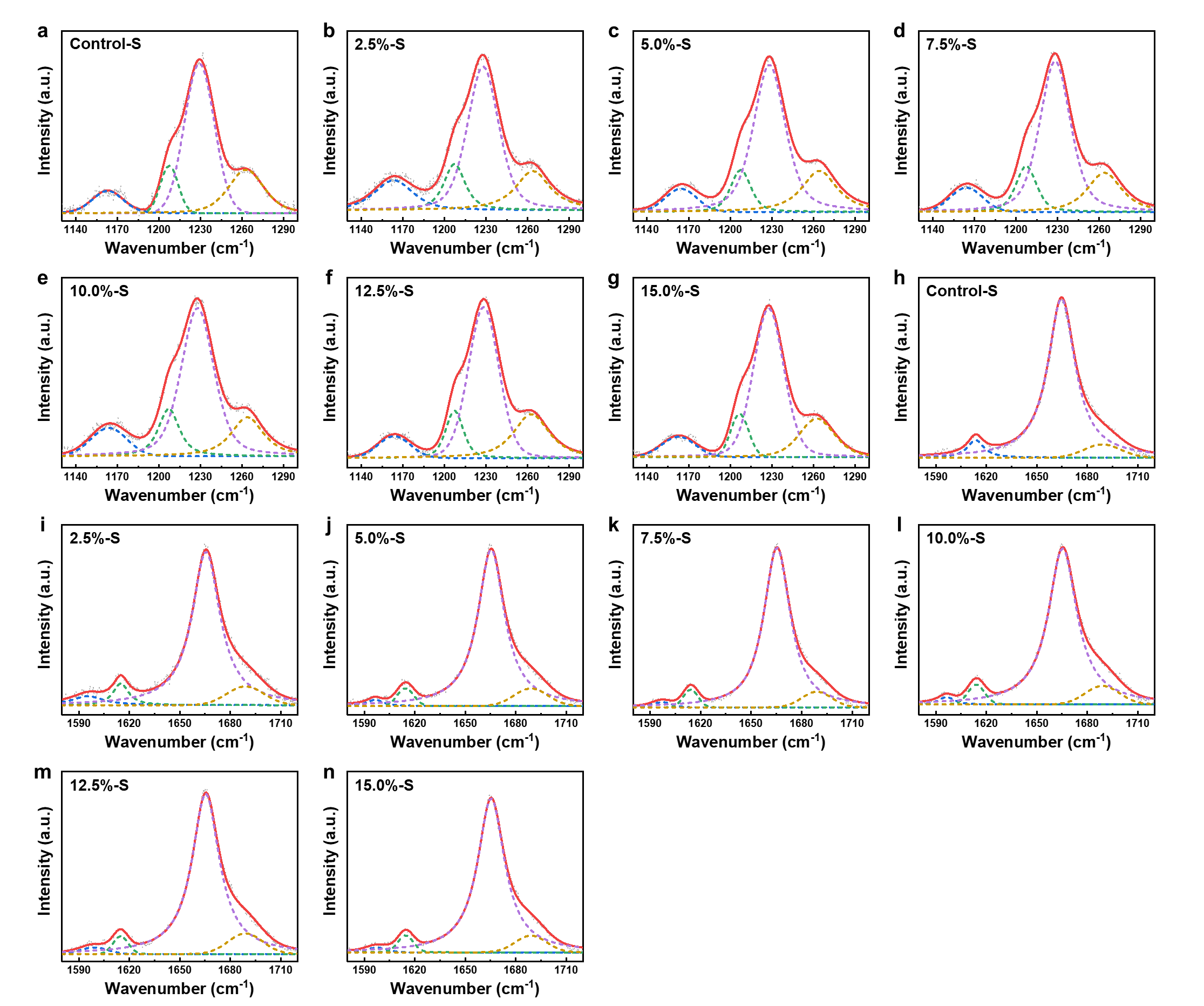


**Figure S9.** Examples showing fitting and deconvolution of Raman spectra. The plots show the original curves (gray, dotted lines), fitting curves (red lines) and deconvolution peaks (blue, green, purple and olive dotted lines). Peak fitting of Raman spectra from 1130 cm^-1^ to 1300 cm^-1^ of Control-S (a), 2.5%-S (b), 5.0%-S (c), 7.5%-S (d), 10.0%-S (e), 12.5%-S (f), and 15.0%-S (g), respectively. Peak fitting of Raman spectra from 1580 cm^-1^ to 1720 cm^-1^ of Control-S (h), 2.5%-S (i), 5.0%-S (j), 7.5%-S (k), 10.0%-S (l), 12.5%-S (m), and 15.0%-S (n), respectively.


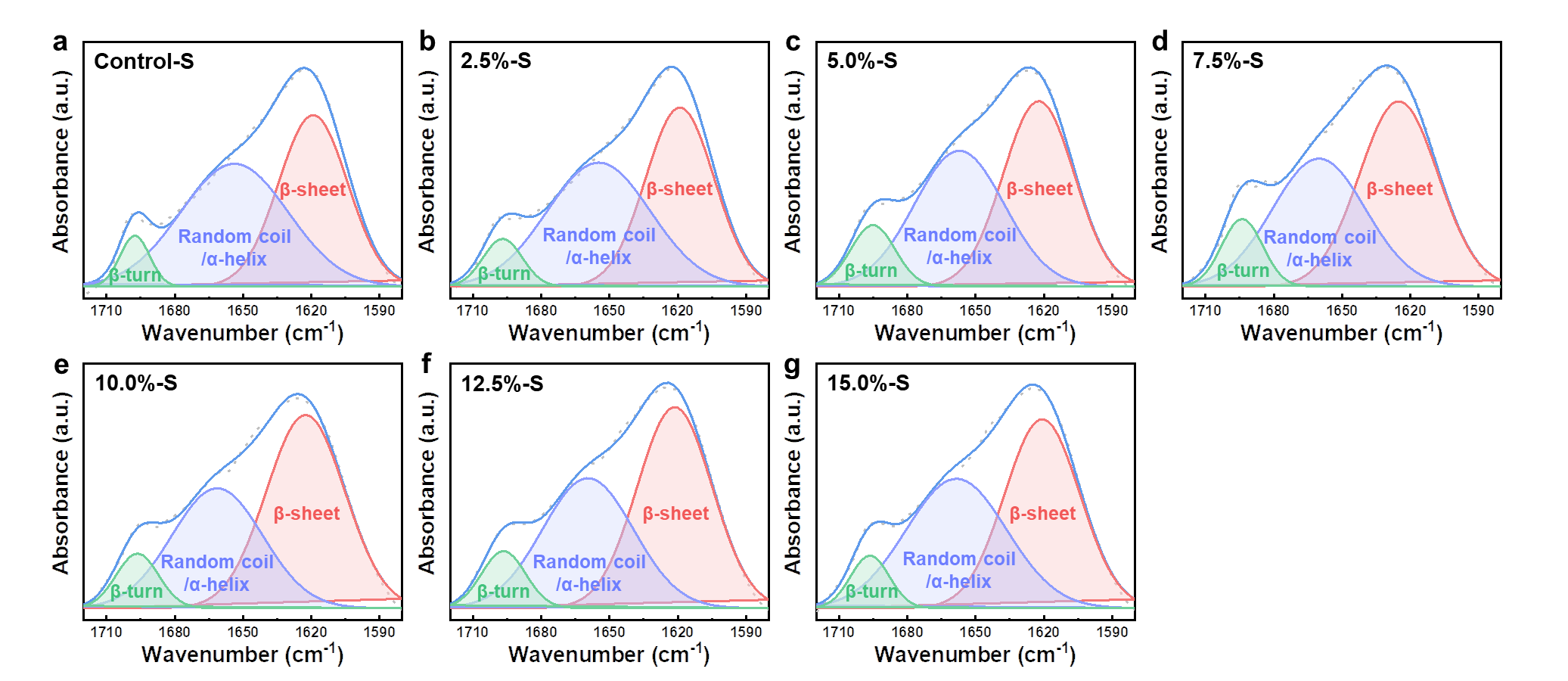


**Figure S10.** Examples showing fitting and deconvolution of amide I band in FTIR spectra. The plots show the original curves (gray, dotted lines), the fitting curves (blue lines), and the deconvolution patterns (green, purple and red areas). Green, purple and red deconvolution patterns refer to the β-turn, random coil/α-helix, and β-sheet structures, respectively. (a) Control-S. (b) 2.5%-S. (c) 5.0%-S. (d) 7.5%-S. (e) 10.0%-S. (f) 12.5%-S. (g) 15.0%-S.

**Table S1.** The standard for classifying the mechanical performance of silk fibers in Figure 1d

| Grade | Modulus  (GPa) | Tensile strength (GPa) | Toughness  (MJ m^-3^) | Elongation at break (%) | Diameter  (µm) |
| --- | --- | --- | --- | --- | --- |
| 1 | 0-6 | 0-0.3 | 0-40 | 0-8 | 0-5 |
| 2 | 6-12 | 0.3-0.6 | 40-80 | 8-16 | 5-10 |
| 3 | 12-18 | 0.6-0.9 | 80-120 | 16-24 | 10-15 |
| 4 | 18-24 | 0.9-0.12 | 120-160 | 24-32 | 15-20 |
| 5 | 24-30 | 0.12-0.15 | 160-200 | 32-40 | 20-25 |

The stiffness and extensibility are evaluated by modulus and elongation at break, respectively.

**Table S2.** Comparison of modulus, tensile strength, toughness and elongation at break of silk fibers

| Silk | Modulus  (GPa) | Tensile strength  (GPa) | Toughness  (MJ m^-3^) | Elongation at break (%) | Diameter  (µm) |
| --- | --- | --- | --- | --- | --- |
| Silkworm SF | 11.0 | 0.6 | 67.8 | 16.4 | 12.1 |
| Spider SF | 10.0 | 1.1 | 160.0 | 27.0 | ~2 |
| HSSF | 21.6 | 0.8 | 79.7 | 12.9 | 10.5 |

SF, silk fibers. AVG, average. MAX, maximum. The mechanical data of Spider SF (*Araneus diadematus* major ampullate (MA) silk) came from reference (1).

**Table S3.** Mechanical properties of silk prepared in Ar and in air with all other conditions the same.

| Atmosphere | Modulus  (GPa) | Tensile strength  (GPa) | Toughness  (MJ m^-3^) | Elongation at break (%) | Diameter  (µm) |
| --- | --- | --- | --- | --- | --- |
| Ar | 13.3±2.1 | 0.59±0.12 | 58.7±11.8 | 14.7±3.0 | 10.1±0.5 |
| Air | 13.8±2.4 | 0.54±0.07 | 53.5±7.1 | 12.5±2.3 | 9.4±0.5 |

**Table S4.** Modulus and tensile strength of regenerated silk and natural silk in Figure 2c

| Name | Young’s Modulus  (GPa) | Tensile strength  (MPa) | Reference |
| --- | --- | --- | --- |
| Silk 1 | 6.7 | 130 | (2) |
|  | 5.3 | 321.2 | (3) |
|  | 5.2 | 193 | (4) |
|  | 4.1 | 103.8 | (5) |
|  | 5.5 | 257.5 | (6) |
|  | 7.2 | 120 | (7) |
|  | 4.9 | 269.4 | (8) |
|  | 5.3 | 127 | (9) |
|  | 4.3 | 400.5 | (10) |
|  | 6 | 128.8 | (11) |
|  | 12.2 | 320 | (12) |
|  | 12.5 | 450 | (13) |
|  | 7.3 | 408 | (14) |
|  | 9.3 | 422 |  |
|  | 9.8 | 295 |  |
|  | 15.2 | 390 | (15) |
|  | 14.2 | 351 | (16) |
|  | 13.7 | 360 |  |
|  | 11.2 | 221 | (17) |
|  | 10.4 | 314 | (18) |
|  | 6.9 | 470.4 | (19) |
|  | 18.9 | 450 | (20) |
|  | 37.8 | 98 | (21) |
|  | 6.9 | 180 | (22) |
|  | 7.2 | 285.1 | (23) |
|  | 13.2 | 550 | (24) |
|  | 7.2 | 120 | (25) |
|  | 5.3 | 127 |  |
|  | 3 | 101 | (26) |
|  | 9 | 250 |  |
|  | 11 | 330 |  |
| Silk 2 | 7.18 | 130 | (27) |
|  | 9.4 | 541.3 | (28) |
|  | 8.8 | 333 | (29) |
|  | 19 | 614 | (30) |
|  | 5.3 | 162.8 | (31) |
|  | 6.8 | 199.2 |  |
|  | 4.8 | 188.5 |  |
|  | 3 | 51.1 |  |
|  | 1.7 | 63.9 | (32) |
|  | 6.4 | 199.8 |  |
|  | 6.2 | 301.5 |  |
|  | 5.8 | 295.2 |  |
|  | 8.8 | 357.3 | (33) |
|  | 11.1 | 337.7 | (34) |
| Silk 3 | 13.2 | 269.6 | (35) |
|  | 1.1 | 49.6 | (36) |
|  | 0.4 | 49.5 | (37) |
|  | 21 | 508 | (38) |
|  | 4.5 | 246.7 | (39) |
|  | 5.7 | 132.5 | (40) |
|  | 4.4 | 127.5 | (41) |
|  | 3.8 | 96.2 |  |
|  | 3.4 | 37.6 | (42) |
|  | 4.3 | 59.6 |  |
|  | 3.9 | 121.9 | (43) |
|  | 2.6 | 95.1 |  |
|  | 4 | 150.6 | (44) |
|  | 9.3 | 308 | (45) |
|  | 2.9 | 53.5 | (46) |
|  | 1.6 | 39 |  |
|  | 3.6 | 46.9 |  |
|  | 8.3 | 192.2 | (47) |
|  | 4 | 370 | (48) |
|  | 4 | 62.3 | (49) |
|  | 8.4 | 286.2 |  |
|  | 6 | 162 | (50) |
|  | 8.0 | 320 | (51) |
|  | 4.6 | 140 | (52) |
|  | 6.3 | 350 | (53) |
| *Araneus diadematus* MA silk | 10 | 1100 | (1) |
| *Araneus diadematus* viscid silk | 0.003 | 500 |  |
| Transgenic silk | 12.9 | 1256.6 | (54) |

Silk 1: regenerated silkworm silk by wet spinning. Silk 2: regenerated silkworm silk by dry spinning. Silk 3: regenerated natural or recombinant spider silk. Some data are directly extract from reference (55, 56).

**References**

1. F. G. Omenetto, D. L. Kaplan, "New opportunities for an ancient material," *Science*, vol. 329, no. 5991, pp. 528-531, 2010.

2. K. Matsumoto, H. Uejima, T. Iwasaki et al., "Studies on regenerated protein fibers. III. Production of regenerated silk fibroin fiber by the self-dialyzing wet spinning method," *Journal of Applied Polymer Science*, vol. 60, no. 4, pp. 503-511, 1996.

3. J. M. Yao, H. Masuda, C. H. Zhao et al., "Artificial spinning and characterization of silk fiber from *Bombyx mori* silk fibroin in hexafluoroacetone hydrate," *Macromolecules*, vol. 35, no. 1, pp. 6-9, 2002.

4. C. H. Zhao, J. M. Yao, H. Masuda et al., "Structural characterization and artificial fiber formation of Bombyx mori silk fibroin in hexafluoro-iso-propanol solvent system," *Biopolymers*, vol. 69, no. 2, pp. 253-259, 2003.

5. I. C. Um, C. S. Ki, H. Y. Kweon et al., "Wet spinning of silk polymer-II. Effect of drawing on the structural characteristics and properties of filament," *International Journal of Biological Macromolecules*, vol. 34, no. 1-2, pp. 107-119, 2004.

6. I. C. Um, H. Y. Kweon, K. G. Lee et al., "Wet spinning of silk polymer-I. Effect of coagulation conditions on the morphological feature of filament," *International Journal of Biological Macromolecules*, vol. 34, no. 1-2, pp. 89-105, 2004.

7. E. Marsano, P. Corsini, C. Arosio et al., "Wet spinning of *Bombyx mori* silk fibroin dissolved in N-methyl morpholine N-oxide and properties of regenerated fibres," *International Journal of Biological Macromolecules*, vol. 37, no. 4, pp. 179-188, 2005.

8. K. H. Lee, D. H. Baek, C. S. Ki et al., "Preparation and characterization of wet spun silk fibroin/poly(vinyl alcohol) blend filaments," *International Journal of Biological Macromolecules*, vol. 41, no. 2, pp. 168-172, 2007.

9. P. Corsini, J. Perez-Rigueiro, G. V. Guinea et al., "Influence of the draw ratio on the tensile and fracture behavior of NMMO regenerated silk fibers," *Journal of Polymer Science Part B-Polymer Physics*, vol. 45, no. 18, pp. 2568-2579, 2007.

10. Z. H. Zhu, K. Ohgo, T. Asakura, "Preparation and characterization of regenerated *Bombyx mori* silk fibroin fiber with high strength," *Express Polymer Letters*, vol. 2, no. 12, pp. 885-889, 2008.

11. S. Sohn, S. P. Gido, "Wet-spinning of osmotically stressed silk fibroin," *Biomacromolecules*, vol. 10, no. 8, pp. 2086-2091, 2009.

12. G. R. Plaza, P. Corsini, E. Marsano et al., "Old silks endowed with new properties," *Macromolecules*, vol. 42, no. 22, pp. 8977-8982, 2009.

13. G. Zhou, Z. Shao, D. P. Knight et al., "Silk Fibers Extruded Artificially from Aqueous Solutions of Regenerated Bombyx mori Silk Fibroin are Tougher than their Natural Counterparts," *Advanced Materials*, vol. 21, no. 3, pp. 366-370, 2009.

14. Z. Zhu, Y. Kikuchi, K. Kojima et al., "Mechanical properties of regenerated *Bombyx mori* silk fibers and recombinant silk fibers produced by transgenic silkworms," *Journal of Biomaterials Science-Polymer Edition*, vol. 21, no. 3, pp. 395-412, 2010.

15. J. Yan, G. Zhou, D. P. Knight et al., "Wet-Spinning of Regenerated Silk Fiber from Aqueous Silk Fibroin Solution: Discussion of Spinning Parameters," *Biomacromolecules*, vol. 11, no. 1, pp. 1-5, 2010.

16. G. R. Plaza, P. Corsini, E. Marsano et al., "Correlation between processing conditions, microstructure and mechanical behavior in regenerated silkworm silk fibers," *Journal of Polymer Science Part B-Polymer Physics*, vol. 50, no. 7, pp. 455-465, 2012.

17. S. Ling, L. Zhou, W. Zhou et al., "Conformation transition kinetics and spinnability of regenerated silk fibroin with glycol, glycerol and polyethylene glycol," *Materials Letters*, vol. 81, no., pp. 13-15, 2012.

18. H. Zhou, Z. Shao, X. Chen, "Wet-spinning of regenerated silk fiber from aqueous silk fibroin solutions: influence of calcium ion addition in spinning dope on the performance of regenerated silk fiber," *Chinese Journal of Polymer Science*, vol. 32, no. 1, pp. 29-34, 2014.

19. F. Zhang, Q. Lu, X. Yue et al., "Regeneration of high-quality silk fibroin fiber by wet spinning from CaCl2-formic acid solvent," *Acta Biomaterialia*, vol. 12, no., pp. 139-145, 2015.

20. G. Fang, Y. Huang, Y. Tang et al., "Insights into silk formation process: correlation of mechanical properties and structural evolution during artificial spinning of silk fibers," *Acs Biomaterials Science & Engineering*, vol. 2, no. 11, pp. 1992-2000, 2016.

21. Z. Chen, H. Zhang, Z. Lin et al., "Programing performance of silk fibroin materials by controlled nucleation," *Advanced Functional Materials*, vol. 26, no. 48, pp. 8978-8990, 2016.

22. R. L. Lock. (Patents, U. S., 1992).

23. C. S. Ki, J. W. Kim, H. J. Oh et al., "The effect of residual silk sericin on the structure and mechanical property of regenerated silk filament," *International Journal of Biological Macromolecules*, vol. 41, no. 3, pp. 346-353, 2007.

24. R. L. Lock. (Patents, U.S., 1993).

25. G. R. Plaza, P. Corsini, J. Perez-Rigueiro et al., "Effect of water on *Bombyx mori* regenerated silk fibers and its application in modifying their mechanical properties," *Journal of Applied Polymer Science*, vol. 109, no. 3, pp. 1793-1801, 2008.

26. R. Madurga, A. M. Ganan-Calvo, G. R. Plaza et al., "Production of high performance bioinspired silk fibers by straining flow spinning," *Biomacromolecules*, vol. 18, no. 4, pp. 1127-1133, 2017.

27. F. Xie, H. Zhang, H. Shao et al., "Effect of shearing on formation of silk fibers from regenerated *Bombyx mori* silk fibroin aqueous solution," *International Journal of Biological Macromolecules*, vol. 38, no. 3-5, pp. 284-288, 2006.

28. Q. Peng, H. Shao, X. Hu et al., "Role of humidity on the structures and properties of regenerated silk fibers," *Progress in Natural Science-Materials International*, vol. 25, no. 5, pp. 430-436, 2015.

29. X. Yue, F. Zhang, H. Wu et al., "A novel route to prepare dry-spun silk fibers from CaCl2-formic acid solution," *Materials Letters*, vol. 128, no., pp. 175-178, 2014.

30. J. Luo, L. Zhang, Q. Peng et al., "Tough silk fibers prepared in air using a biomimetic microfluidic chip," *International Journal of Biological Macromolecules*, vol. 66, no., pp. 319-324, 2014.

31. W. Wei, Y. Zhang, Y. Zhao et al., "Studies on the post-treatment of the dry-spun fibers from regenerated silk fibroin solution: Post-treatment agent and method," *Materials & Design*, vol. 36, no., pp. 816-822, 2012.

32. W. Wei, Y. Zhang, H. Shao et al., "Posttreatment of the dry-spun fibers obtained from regenerated silk fibroin aqueous solution in ethanol aqueous solution," *Journal of Materials Research*, vol. 26, no. 9, pp. 1100-1106, 2011.

33. Y. Jin, Y. Zhang, Y. Hang et al., "A simple process for dry spinning of regenerated silk fibroin aqueous solution," *Journal of Materials Research*, vol. 28, no. 20, pp. 2897-2902, 2013.

34. M. Sun, Y. Zhang, Y. Zhao et al., "The structure-property relationships of artificial silk fabricated by dry-spinning process," *Journal of Materials Chemistry*, vol. 22, no. 35, pp. 18372-18379, 2012.

35. A. Lazaris, S. Arcidiacono, Y. Huang et al., "Spider silk fibers spun from soluble recombinant silk produced in mammalian cells," *Science*, vol. 295, no. 5554, pp. 472-476, 2002.

36. F. Teule, W. A. Furin, A. R. Cooper et al., "Modifications of spider silk sequences in an attempt to control the mechanical properties of the synthetic fibers," *Journal of Materials Science*, vol. 42, no. 21, pp. 8974-8985, 2007.

37. A. E. Brooks, S. M. Stricker, S. B. Joshi et al., "Properties of synthetic spider silk fibers based on *Argiope aurantia* MaSp2," *Biomacromolecules*, vol. 9, no. 6, pp. 1506-1510, 2008.

38. X. Xia, Z. Qian, C. S. Ki et al., "Native-sized recombinant spider silk protein produced in metabolically engineered Escherichia coli results in a strong fiber," *Proceedings of the National Academy of Sciences of the United States of America*, vol. 107, no. 32, pp. 14059-14063, 2010.

39. M. Elices, G. V. Guinea, G. R. Plaza et al., "Bioinspired fibers follow the track of natural spider silk," *Macromolecules*, vol. 44, no. 5, pp. 1166-1176, 2011.

40. B. An, M. B. Hinman, G. P. Holland et al., "Inducing beta-sheets formation in synthetic spider silk fibers by aqueous post-spin stretching," *Biomacromolecules*, vol. 12, no. 6, pp. 2375-2381, 2011.

41. F. Teule, B. Addison, A. R. Cooper et al., "Combining flagelliform and dragline spider silk motifs to produce tunable synthetic biopolymer fibers," *Biopolymers*, vol. 97, no. 6, pp. 418-431, 2012.

42. B. An, J. E. Jenkins, S. Sampath et al., "Reproducing natural spider silks’ copolymer behavior in synthetic silk mimics," *Biomacromolecules*, vol. 13, no. 12, pp. 3938-3948, 2012.

43. E. Gnesa, Y. Hsia, J. L. Yarger et al., "Conserved C-terminal domain of spider tubuliform spidroin 1 contributes to extensibility in synthetic fibers," *Biomacromolecules*, vol. 13, no. 2, pp. 304-312, 2012.

44. S. L. Adrianos, F. Teule, M. B. Hinman et al., "*Nephila clavipes* flagelliform silk-like GGX motifs contribute to extensibility and spacer motifs contribute to strength in synthetic spider silk fibers," *Biomacromolecules*, vol. 14, no. 6, pp. 1751-1760, 2013.

45. Z. Lin, Q. Deng, X. Liu et al., "Engineered large spider eggcase silk protein for strong artificial fibers," *Advanced Materials*, vol. 25, no. 8, pp. 1216-1220, 2013.

46. A. E. Albertson, F. Teule, W. Weber et al., "Effects of different post-spin stretching conditions on the mechanical properties of synthetic spider silk fibers," *Journal of the Mechanical Behavior of Biomedical Materials*, vol. 29, no., pp. 225-234, 2014.

47. J. A. Jones, T. I. Harris, C. L. Tucker et al., "More than just fibers: an aqueous method for the production of innovative recombinant spider silk protein materials," *Biomacromolecules*, vol. 16, no. 4, pp. 1418-1425, 2015.

48. A. Heidebrecht, L. Eisoldt, J. Diehl et al., "Biomimetic fibers made of recombinant spidroins with the same toughness as natural spider silk," *Advanced Materials*, vol. 27, no. 13, pp. 2189-2194, 2015.

49. Q. Peng, Y. Zhang, L. Lu et al., "Recombinant spider silk from aqueous solutions via a bio-inspired microfluidic chip," *Scientific Reports*, vol. 6, no., pp., 2016.

50. M. Andersson, Q. Jia, A. Abella et al., "Biomimetic spinning of artificial spider silk from a chimeric minispidroin," *Nature Chemical Biology*, vol. 13, no. 3, pp. 262-264, 2017.

51. A. Seidel, O. Liivak, S. Calve et al., "Regenerated spider silk:  processing, properties, and structure," *Macromolecules*, vol. 33, no. 3, pp. 775-780, 2000.

52. S. R. Fahnestock. (E. I. du Pont de Nemours and Company, 2001).

53. M. Elices, G. V. Guinea, G. R. Plaza et al., "Bioinspired fibers follow the track of natural spider silk," *Macromolecules*, vol. 44, no. 5, pp. 1166-1176, 2011.

54. Q. Wang, S. Ling, X. Liang et al., "Self‐Healable Multifunctional Electronic Tattoos Based on Silk and Graphene," *Advanced Functional Materials*, vol. 29, no. 16, pp., 2019.

55. A. Koeppel, C. Holland, "Progress and trends in artificial silk spinning: a systematic review," *Acs Biomaterials Science & Engineering*, vol. 3, no. 3, pp. 226-237, 2017.

56. S. Ling, Z. Qin, C. Li et al., "Polymorphic regenerated silk fibers assembled through bioinspired spinning," *Nature Communications*, vol. 8, no., pp. 1387, 2017.
